# Effect of thoracic paravertebral block with ropivacaine and compound betamethasone on postoperative pain after thoracoscopic surgery: A randomized, double-blind, controlled trial

**DOI:** 10.1097/MD.0000000000046573

**Published:** 2025-12-26

**Authors:** Mengru Cui, Dingying Ge, Qing She, Yibin Qin, Cui’e Lu

**Affiliations:** a Department of Anesthesiology, Affiliated Hospital of Nantong University, Nantong, China; b Department of Anesthesiology, Yancheng Third People’s Hospital, Yancheng, China.

**Keywords:** compound betamethasone, postoperative pain, ropivacaine, TPVB

## Abstract

**Background:**

The purpose was to evaluate the effect of thoracic paravertebral block (TPVB) with a combination of ropivacaine and compound betamethasone on postoperative pain in patients undergoing thoracoscopic surgery.

**Methods:**

A randomized, double-blind, controlled trial was conducted. A total of 100 patients undergoing elective thoracoscopic pulmonary lesion resection were randomly divided into 2 groups: the ropivacaine group (group R) and the ropivacaine combined with compound betamethasone group (group RD). The pain intensity at rest/coughing was assessed by the Visual Analogue Scale (VAS) at different times. The incidence of pain (VAS ≥ 1) at 1-, 3-, and 6-month follow-up was recorded. The anesthetic dosage, adverse effects, and postoperative recovery parameters were also observed.

**Results:**

Compared to group R, group RD demonstrated a significant reduction in additional patient-controlled intravenous analgesia pump presses at 24 hours postoperatively and significantly lower VAS scores at rest/coughing after extubation, and at 6, 12, 24, 48, 72 hours, and 1-month postoperatively (*P* <.05). Group RD reduced the incidence of pain at 1, 3, and 6 months postoperatively more than group R (*P* <.05), as well as in postoperative nausea, vomiting, and drowsiness. No statistically significant differences were observed between the 2 groups regarding postoperative blood glucose levels, duration of drainage tube placement, time to first mobilization, and length of hospital stay.

**Conclusion:**

These results suggest that the combination of ropivacaine and compound betamethasone in TPVB effectively reduces both acute and chronic postoperative pain after thoracoscopic surgery. It also decreases the incidence of postoperative adverse reactions, making it a superior adjuvant for TPVB with local anesthetics.

## 1. Introduction

Thoracoscopic surgery, as a minimally invasive procedure in thoracic surgery, despite relatively low trauma, has an incidence of chronic postoperative pain (CPSP) as high as 40% to 60%. 31.5% of the CPSP is neuropathic pain, which is correlated with the degree of acute postoperative pain.^[1,2]^ Commonly used opioids may carry the risk of addiction and respiratory depression, while nonsteroidal anti-inflammatory drugs can cause gastrointestinal reactions. Therefore, it is extremely urgent for us to optimize postoperative pain management.

Enhanced recovery after surgery guidelines for thoracic surgery recommend a multimodal approach to pain management, thereby reducing the use of opioids. They advocate the use of different analgesic drugs or regional anesthesia.^[3]^ Thoracic epidural anesthesia has been the gold standard for preventing postoperative pain in thoracic surgery.^[4]^ However, it has disadvantages such as high coagulation requirements, respiratory depression, circulatory instability, epidural hematoma, and total spinal anesthesia. TPVB has become a new trend in analgesia for thoracic surgery in recent years.^[5,6]^ TPVB improves postoperative analgesia and enhances patients’ quality of life. It is recognized as a superior technique for regional anesthesia.^[7]^ Local anesthetics (LAs) are widely used in pain management by blocking the reversible transmission of peripheral nerve signals. However, their analgesic duration is short.^[8]^ Studies have shown that combining LAs with adjuvants can optimize postoperative pain management.^[9]^ Dexamethasone as an adjuvant to ropivacaine for paravertebral block is effective in prolonging analgesia.^[10]^ Compound betamethasone, a synthetic glucocorticoid, significantly reduces pain in tendinitis and arthritis of the long head of the biceps brachii when used topically.^[11]^ However, there has been no detailed investigation of the effect of compound betamethasone as an adjuvant on the acute and chronic pain after thoracic surgery. The aim of this study was to evaluate this effect and to provide a reference for clinical application.

## 2. Methods

### 2.1. General information

Patients who underwent elective thoracoscopic pulmonary lesion resection from May to December 2021 in our hospital were selected, regardless of gender, aged 18 to 70 years, BMI: 18.5 to 28 kg/m², and classified as American Society of Anesthesiologists I-III.

### 2.2. Exclusion criteria

Refusal to participate, cognitive dysfunction, infection at the puncture site, severe coagulation dysfunction, serious cardiac, pulmonary, hepatic, or renal insufficiency, allergy to LAs or glucocorticoids, long-term preoperative use of opioids, history of thoracic surgery, presence of central nervous system (CNS) disease or peripheral neuropathy, and other conditions that require caution in the use of glucocorticoids, such as bone fracture, osteoporosis, trauma repair period, corneal ulcers, hyperadrenocorticism, diabetes mellitus, pregnancy, etc. Additionally, patients requiring postoperative reoperation or admission to the intensive care unit, those with serious complications threatening life, or those who failed to complete the VAS score and telephone follow-up as required were excluded.

### 2.3. Ethical approval

All the patients and their families gave their written informed consent after they had received a detailed explanation of the research project. They were informed that they had an optional withdrawal at any time. This study was a randomized, double-blind, controlled trial. It was performed in accordance with the human subjects’ protection principles (Declaration of Helsinki). Ethical approval was granted by the Ethics Committee of Affiliated Hospital of Nantong University in China (May 20, 2021, 2021 – k047 – 01). The study was registered on ClinicalTrials.gov (January 3, 2022, NCT05175001).

### 2.4. Grouping and treatment

Patients were divided into 2 groups using a randomized numerical allocation method: the ropivacaine group (Group R) and the compound betamethasone combined with ropivacaine group (Group RD). The experimental protocol number for each subject was generated by a computer-generated random assignment sequence and placed in a sealed, opaque envelope. Eligible subjects opened the envelopes in sequence and were grouped as determined by the anesthesia regimen in the envelope. The entire grouping process was performed by a staff member not involved in the experiment, and neither the investigator nor the subjects were aware of the specific grouping.

**Figure 1.**
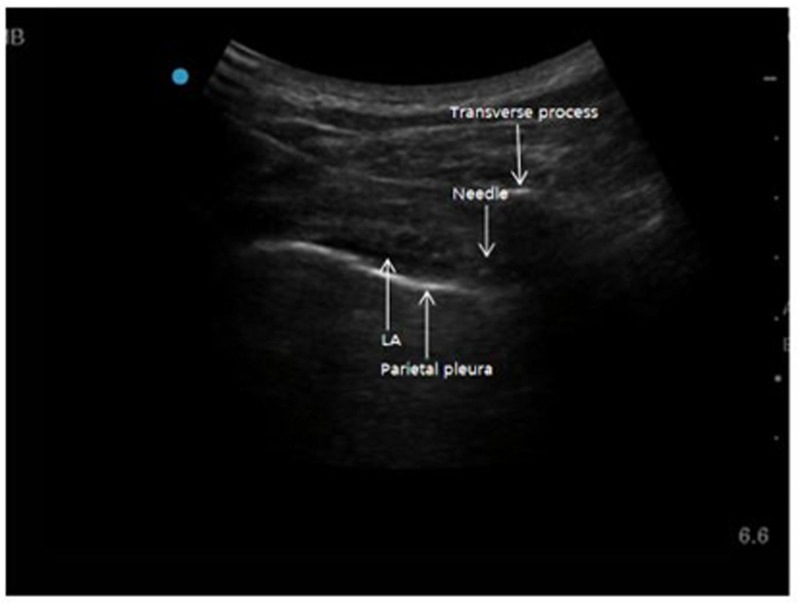
TPVB under ultrasound guidance. The high echo area is transverse process. The strip of hyperechoic zone is parietal pleura. The free fluid area is LA. The pleura is pressed down obviously after injection of LA. LA = local anesthetic.

After the patient was intubated with a double-lumen tube induced by general anesthesia, the patient was placed in the lateral position with the operative side facing upward, and an ultrasound-guided thoracic paravertebral nerve block was performed by selecting 3 vertebral interspaces on the operative side, namely, T4–5, T6–7, and T8-9. After routine disinfection with iodine-vapor and placing a sterile cavity towel, an ultrasound probe (SONOSITE EDGE II, C60/5-2 MHz, USA) was positioned at the level of the transverse process of the target segment (frequency of 2–5 MHz), scanning the area near the puncture point, locating the transverse costovertebral joints, and then moving caudolaterally towards the ribs until the ribs disappeared, revealing only the transverse process. An in-plane approach in the paravertebral transverse section was utilized, with the transverse process serving as a landmark. A 22G puncture needle (0.7 × 80 TWLB; KDL Medical Devices Inc, Zhejiang, China) was inserted inwardly on the lateral side of the probe until the tip reached below the lateral end of the transverse process, where the intercostal space transitions to the thoracic paravertebral space. The needle was then retracted to ensure no blood, gas, or cerebrospinal fluid was present, and pleural sub-pressure was observed after injecting a small amount of medication (Fig. 1), confirming correct needle placement. Group R: Injection of 0.33% ropivacaine, 10 mL in each paravertebral space, totaling 30 mL. Group RD: Injection of 4.67 mg of compound betamethasone combined with 0.33% ropivacaine, 10 mL in each paravertebral space, totaling 30 mL. The procedures were performed by the same attending anesthesiologist who had completed the necessary training.

### 2.5. Anesthesia methods

After admission, peripheral venous access was routinely established, and noninvasive blood pressure (NIBP), heart rate, electrocardiogram (ECG), and peripheral oxygen saturation (SpO₂) were continuously monitored. Radial artery puncture was performed under local anesthesia with 1% lidocaine, followed by intubation, and invasive blood pressure monitoring was initiated. Induction of anesthesia was achieved by administering Midazolam (0.05 mg/kg), Propofol (1.5 mg/kg), Sufentanil (0.5 μg/kg), and Cisatracurium (0.2 mg/kg) intravenously in sequence. After the onset of muscle relaxation, double-lumen bronchial intubation was performed orally, and mechanical ventilation was initiated following fiberoptic bronchoscopy placement. Ventilation was maintained in volume-controlled mode with 2-lung ventilation, FiO₂ set at 80%, oxygen flow at 1 to 2 L/min, tidal volume (VT) of 6 to 8 mL/kg, and respiratory rate of 10 to 12 breaths/min. Five minutes before skin incision, ventilation was switched to 1-lung ventilation with VT adjusted to 5 to 6 mL/kg and respiratory rate to 12 to 16 breaths/min. Peak airway pressure (Ppeak) was kept below 30 cmH₂O, and end-tidal CO₂ (P_ET_CO₂) was maintained between 35 to 45 mm Hg. Respiratory parameters were adjusted to maintain SpO₂ above 94% and P_ET_CO₂ within 35 to 45 mm Hg.

Anesthesia was maintained with Sevoflurane (1–2 vol%), Propofol (2–4 mg/kg/h), Remifentanil (0.1–0.2 μg/kg/min), and Cisatracurium (0.1–0.2 mg/kg/h) via intravenous infusion. Medications were adjusted to maintain a Bispectral Index (BIS) of 40 to 60. Norepinephrine was administered to maintain blood pressure within ± 20% of the basal value. Intravenous Atropine (0.3–0.5 mg) was administered when heart rate was ≤ 45 beats/min. An additional 10 μg of Sufentanil was administered as needed.

Postoperatively, patients were uniformly transferred to the post-anesthesia care unit, and the endotracheal tube was removed once spontaneous respiration was restored and consciousness was regained. After stabilization of vital signs and achieving a Steward awakening score > 4, patients were returned to the ward.

Both groups were connected to patient-controlled intravenous analgesia (PCIA) pumps postoperatively. The PCIA solution consisted of 150 μg Sufentanil and 15 mg dezocine diluted in 100 mL of 0.9% sodium chloride solution. The PCIA parameters were set as follows: an infusion rate of 2 mL/h, a self-control dose of 0.5 mL/dose, and a lockout time of 10 minutes. When the Visual Analog Scale (VAS) score exceeded 3, the self-control dose was administered as a remedial analgesic measure.

#### Additional measures

If the VAS score remained above 3 after 2 consecutive PCIA compressions, dezocine 5 mg was administered as additional analgesia. In cases of postoperative nausea and vomiting, Azasetron 10 mg was administered intravenously.

### 2.6. Observational indexes

Primary outcomes:

1. Assess resting and motion (cough) pain scores using the Visual Analog Scale (VAS) at preoperative, post-extubation, and at 6, 12, 24, 48, 72 hours, and 1-month postoperatively.
2. Record the incidence of pain (VAS ≥ 1) at 1-month, 3-months, and 6 months postoperatively.
3. Measure the use of PCIA pumps within the first 24 hours postoperatively, specifically the total number of additional pump presses with VAS scores > 3.

Secondary Outcomes:

1. Record intraoperative dosages of Sufentanil, Remifentanil, and Norepinephrine, as well as the duration of 1-lung ventilation and surgery.
2. Measure patients’ fasting blood glucose levels preoperatively, postoperatively, and at 72 hours postoperatively.
3. Document adverse reactions such as postoperative nausea, vomiting, and drowsiness, as well as the incidence of pulmonary complications.
4. Record postoperative recovery parameters including duration of drain placement, time to first mobilization, and length of hospital stay.

### 2.7. Statistical analysis

#### Sample size calculation

Based on the results of a study by Mao et al.^[12]^ on acute postoperative pain in thoracic patients, the standard deviations of the number of additional analgesic pump presses were 9.88 for the control group and 5.35 for the 0.5% ropivacaine TPVB group at 72 hours postoperatively. Using PASS 15.0 software (NCSS, LLC, Kaysville, Utah), with a confidence level of 90% and a precision of 3, the required sample size was calculated to be 40 cases per group. Considering an approximate 20% loss to follow-up rate, the final number of patients included in each group was determined to be 50, totaling 100 cases.

Statistical analysis was performed using SPSS 25.0 (SPSS Inc., USA) software. Normally distributed measurements were expressed as mean ± standard deviation (x̄ ± s), and repeated measures ANOVA was used for intragroup comparisons. Independent samples t-tests were used for inter-group comparisons. Categorical data were expressed as numbers (%) and compared using the χ² test or Fisher exact test as appropriate. A *P*-value of < 0.05 was considered statistically significant.

### 2.8. Data availability

The data that supports the fundings of this research is available from the corresponding author. The data is not publicly available due to privacy or ethical restrictions.

## 3. Results

### Clinical characteristics

**Figure 2.**
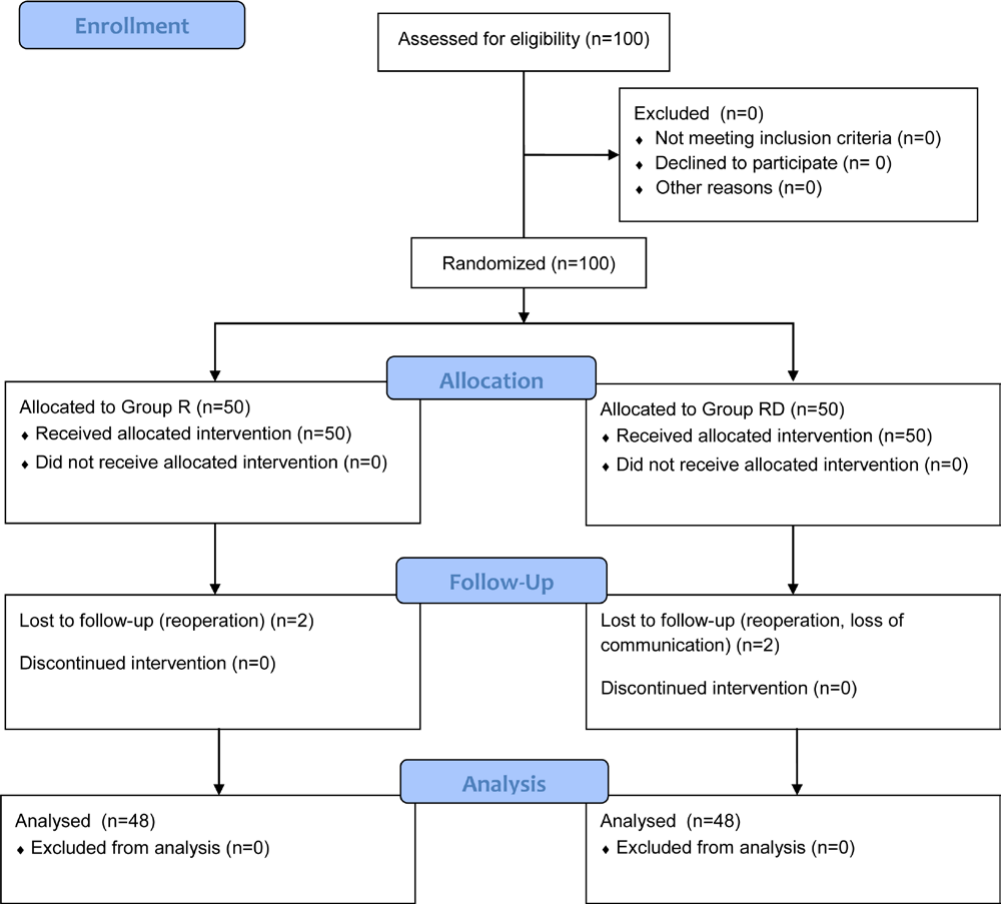
Flow diagram.

The flow diagram of this research process is shown in Figure 2. Initially, 100 patients were enrolled into this study. Four patients were excluded due to postoperative reoperation (n = 3) and loss to follow-up (n = 1), respectively. Finally, 96 patients were included in the analysis (n = 48 in each group).

There were no statistically significant differences between the 2 groups in terms of gender, age, height, BMI, American Society of Anesthesiologists classification, operation time, single-lung ventilation time, and extubation time (Table 1).

**Table 1:** Demographic and clinical characteristics in 2 groups. Values are expressed as the number of participants or mean ± standard deviation (x̄ ± s).

Demographics	Group R(n = 48)	Group RD(n = 48)	*P*-value
Sex (female/male)	23/25	25/23	.68
Age(years)	58.00 ± 7.24	57.40 ± 7.78	.66
Height (cm)	164.69 ± 7.22	165.27 ± 7.67	.70
Weight (kg)	63.13 ± 9.43	62.31 ± 11.32	.79
BMI (kg/m^2^)	23.20 ± 2.55	22.80 ± 3.65	.59
ASA II/ III	42/6	43/5	.75
operation time (h)	1.92 ± 0.58	1.85 ± 0.76	.47
single-lung ventilation time (h)	1.63 ± 0.55	1.60 ± 0.71	.80
extubation time (min)	14.19 ± 6.33	14.31 ± 8.65	.54

ASA = American Society of Anesthesiologists, BMI = body mass index.

### 3.1. Intraoperative and postoperative pharmacological management

Compared with group R, group RD had a significantly lower number of additional PCIA pump presses in the 24-hour postoperative period (*P* < .05). There were no statistically significant differences in the use of intraoperative analgesics Sufentanil and Remifentanil, or the vasoactive drug Norepinephrine between the 2 groups (Table 2).

**Table 2:** Intraoperative and postoperative pharmacological management in 2 groups. Values are expressed as the mean ± standard deviation (x̄ ± s).

	Sufentanil (ug)	Remifentanil (mg)	Norepinephrine (mg)	additional PCIA pump presses (n)
Group R	40.83 ± 5.09	1.17 ± 0.32	0.41 ± 0.20	8.31 ± 2.11
Group RD	41.67 ± 5.77	1.09 ± 0.40	0.38 ± 0.22	3.77 ± 1.82*

PCIA = patient-controlled intravenous analgesia.

Compared to Group R, * *P* < .05.

#### Pain intensity

The difference in preoperative VAS scores between the 2 groups was not statistically significant. Compared with Group R, VAS scores at rest/coughing were significantly lower in Group RD after extubation, and at 6, 12, 24, 48, 72 hours, and 1-month postoperatively (*P* < .05). VAS scores in Group R gradually increased postoperatively, peaked at 12 hours, then gradually decreased, and increased again at 1-month postoperatively (*P* < .05). The trend of changes in VAS at rest in Group RD was consistent with that of Group R, but VAS scores at coughing peaked at 24 hours and were significantly lower at 1-month postoperatively (*P* < .05) (Table 3).

**Table 3:** Pain intensity (VAS at rest/coughing) outcome data. Values are expressed as the mean ± standard deviation (x̄ ± s).

Variable	VAS at rest		VAS at coughing	
	Group R	Group RD	Group R	Group RD
VAS pre	0	0	0.04 ± 0.28	0
VAS extubation	1.13 ± 1.28	0.59 ± 0.84*	1.64 ± 1.65	1.00 ± 1.05*
VAS at 6 h	1.31 ± 0.98	0.58 ± 0.81*	2.41 ± 1.13	1.22 ± 1.03*
VAS at 12 h	0.58 ± 0.81 ^a^	1.04 ± 1.18*^,^**	4.00 ± 1.34**	1.73 ± 1.41*
VAS at 24 h	1.67 ± 0.82	0.63 ± 0.78*	2.81 ± 1.04	1.99 ± 0.71*,**
VAS at 48 h	1.18 ± 0.99	0.18 ± 0.53*	2.53 ± 1.08	1.67 ± 0.59*
VAS at 72 h	0.60 ± 0.82	0.09 ± 0.37*	2.05 ± 1.09	1.38 ± 0.75*
VAS at 1 mo	1.60 ± 1.03 ^b^	0.58 ± 1.05*,**	2.52 ± 1.16**	0.94 ± 1.39*

Compared to Group R, * *P* < .05.

Compared to the previous time point, ** *P* < .05.

#### The incidence of postoperative pain at 1, 3, and 6 months

The incidence of pain at 1, 3, and 6 months postoperatively was significantly lower in the RD group compared with the R group (*P* < .05) (Table 4).

**Table 4:** The incidence of postoperative pain at 1, 3, and 6 months. Values are expressed as the number of participants (percentage).

	1 mo (%)	3 mo (%)	6 mo (%)
Group R	38 (79.17)	25 (52.08)	14 (29.17)
Group RD	13 (27.08)*	10 (20.83)*	6 (12.50)*

Compared to Group R, * *P* < .05.

#### Changes of fasting blood glucose levels

There were no statistically significant differences in preoperative, postoperative, and 72-hour postoperative fasting blood glucose levels between the 2 groups (Table 5).

**Table 5:** The changes of fasting blood glucose levels at preoperative, postoperative, and 72-hour postoperative. Values are expressed as the number of participants (percentage).

	Preoperative	Postoperative	72-h postoperative
Group R	38 (79.17)	25 (52.08)	14 (29.17)
Group RD	13 (27.08)*	10 (20.83)*	6 (12.50)*

Compared to Group R, * *P* < .05.

#### Adverse reactions

The incidence of postoperative nausea, vomiting, and drowsiness was significantly lower in group RD compared with group R (*P* < .05). No cases of postoperative urinary retention, pruritus, hypoxemia, or pulmonary complications occurred in either group (Table 6).

**Table 6:** The adverse reactions in 2 groups. Values are expressed as the number of participants (percentage).

	Nausea/vomiting	Drowsiness	Urinary retention	Pruritus	Hypoxemia	Pulmonary complications
Group R	27 (56.25)	21 (43.75)	0 (0)	0 (0)	0 (0)	0 (0)
Group RD	14 (29.17)*	10 (20.83)*	0 (0)	0 (0)	0 (0)	0 (0)

Compared to Group R, * *P* < .05.

#### Postoperative recovery:

There were no statistically significant differences in the duration of postoperative drain placement (chest tube to water seal bottle, negative pressure balloon), time to first bed movement, and length of hospitalization between the 2 groups (Table 7).

**Table 7:** Postoperative recovery of 2 groups. Values are expressed as the mean ± standard deviation (x̄ ± s).

	Water seal bottle (d)	Negative pressure balloon (d)	Time to first bed movement (d)	Length of hospitalization (d)
Group R	1.35 ± 0.70	3.56 ± 0.50	1.40 ± 0.49	3.63 ± 0.49
Group RD	1.50 ± 1.07	3.48 ± 0.85	1.31 ± 0.51	3.52 ± 0.83

## 4. Discussion

The incidence and severity of acute and chronic pain in postoperative thoracic surgery patients are higher compared to other surgical procedures.^[13]^ Postoperative pain limits the recovery of coughing, sputum clearance, and respiratory function, and in severe cases, can lead to pneumonia, pulmonary atelectasis, and bronchospasm.^[14]^ TPVB provides effective analgesia after thoracic surgery, with fewer side effects and relative hemodynamic stability.^[15,16]^ Regional anesthetic analgesia attenuates the immunosuppression caused by surgical stress, opioids, and narcotics.^[17,18]^ TPVB may attenuate central sensitization by reducing neuroinflammation and may treat thoracic postoperative pain by modulating dorsal root ganglia and spinal cord pain signaling.^[19]^

Thoracoscopic lung-occupying resection incisions involving the T4–5 to T7–8 intercostal spaces require blockade of more than 4 dermatomes to achieve adequate analgesia; a single incision block can still result in severe postoperative pain.^[20]^ The diffusion effect of a single, single-site injection of a large volume of LA is unpredictable. In this study, 3 vertebral spaces – T4–5, T6–7, and T8–9 – were selected for multipoint puncture blocks, which can fully cover the surgical area and achieve satisfactory analgesia. Ropivacaine is a long-acting amide LA that produces reversible blockade of nerve impulse conduction by inhibiting sodium ion influx in the nerve fiber cell membrane. It has been widely used in paravertebral nerve blocks to alleviate acute postoperative pain.^[21,22]^ Compared with 0.25% ropivacaine, 0.33% and 0.5% ropivacaine for TPVB significantly reduced VAS scores within 24 hours after thoracic surgery; however, there was no significant advantage of 0.5% ropivacaine over 0.33% ropivacaine.^[23]^ To minimize the impact of LAs on intraoperative hemodynamics, a relatively low effective concentration dose of 0.33% was chosen in this study.

Single-dose ropivacaine applied to TPVB has a limited duration of action and cannot provide long-lasting analgesia. The clinical use of glucocorticoids remains controversial, but their benefits and safety in regional block analgesia have been demonstrated.^[24]^ Glucocorticoids (e.g., compound betamethasone, methylprednisolone, and dexamethasone) have recently been used as adjuvants to LAs in paravertebral nerve blocks for the treatment of various clinical acute and chronic pain conditions.^[25,26]^ Dexamethasone combined with ropivacaine TPVB did not reduce the incidence of CPSP in patients undergoing thoracic surgery.^[27]^ Compound betamethasone is a mixture of betamethasone sodium phosphate and betamethasone dipropionate. The low water-soluble betamethasone dipropionate in it is excreted over more than 10 days and exerts a long-lasting effect through slow absorption.^[27]^ According to the clinical instructions for the use of compound betamethasone and the range of safe doses for its perineural administration reported in the relevant literature,^[28]^ and combined with the experience of using compound betamethasone to treat relevant chronic neuropathic pain in the pain department of our hospital, we chose 4.67 mg as the dosage for the experimental group in this trial. During the study, no cases of hyperglycemia, delayed wound healing, or wound infection were observed in the RD group.

The underlying pathophysiological process of chronic pain after thoracic surgery is complex and is mainly divided into 2 major mechanisms: the peripheral and CNSs, involving interactions between the immune and nervous systems.^[29]^ Betamethasone and other glucocorticoids can cross the blood-brain barrier to reduce CNS inflammation or oxidative stress, inhibit neuronal apoptosis, modulate synaptic plasticity, and improve neurological function.^[30]^ Local application of betamethasone to damaged nerves can inhibit astrocyte activation and the release of inflammatory mediators in a rat neuropathic pain model.^[31]^ Local application of betamethasone after spinal nerve root compression reduces the expression of substance P in dorsal root ganglia as well as CD4- and CD5-labeled lymphocytes at the site of nerve root compression.^[32]^ Perineural injection of compound betamethasone promotes the recovery of damaged nerve structure and function. The degree of acute postoperative pain is a significant risk factor for the development of CPSP after thoracic surgery.^[33]^ Transient inflammation in the subacute phase after tissue incision can induce reactivation of injury perception and cause long-term injury hypersensitivity.^[34]^ The postoperative subacute phase may also be a critical period for the transition from acute postoperative pain to persistent pain.

The present study showed that VAS scores in the RD group were significantly lower than those in the R group during the acute postoperative period (within 72 hours). This suggests that compound betamethasone may reduce postoperative pain by rapidly increasing the plasma concentration of the active metabolite of betamethasone sodium phosphate, thereby inhibiting inflammatory mediators, glial cell activation, attenuating neuroedema, and decreasing spontaneous ectopic discharges of damaged nerves, sympathetic sprouting, and central sensitization.^[30]^ Watanabe et al.^[28]^ demonstrated that ropivacaine combined with compound betamethasone... This study showed that the VAS score peaked at 12 hours after surgery in the R group and 24 hours after surgery in the RD group during the acute phase, and the analgesic effect was prolonged up to 12 hours. This may be related to the slow release of the low water-soluble betamethasone dipropionate in compound betamethasone, which has a longer-lasting effect on the peripheral nerves. The resting VAS scores of Group R were significantly higher than those of Group RD at 72 hours and 3 months after surgery, suggesting that secondary injury and/or inflammation may have occurred during the subacute phase after thoracic surgery, inducing the reactivation of injury sensation and facilitating the transformation of acute pain to chronic pain. The incidence of pain in Group RD was significantly lower than in Group R at 1 month, 3 months, and 6 months after surgery, suggesting that effective pain control by compound betamethasone may have a significant effect on the acute phase of postoperative pain in thoracic surgery. Effective control of pain in the acute phase may also be a factor in reducing the occurrence of CPSP.

The results of this study showed that the RD group had a significant reduction in the number of additional postoperative PCIA pump compressions, a reduction in the cumulative use of Sufentanil and Diazoxide per unit of time, and a significant reduction in the overall incidence of nausea, vomiting, and somnolence compared with the R group. Based on the fact that patients in the RD group had better postoperative analgesia and fewer adverse effects, we believe that compound betamethasone is a superior adjuvant for LA TPVB to suppress acute postoperative pain.

This study has some limitations. First, all patients underwent TPVB after induction of general anesthesia, making it impossible to assess the block’s effectiveness by testing the patients’ sensory planes. Considering the use of ultrasound guidance, the success rate of ultrasound scanning through the transverse process of the spine and the pleural wall layers under direct vision can be as high as 94%,^[35]^ and all procedures were performed by an experienced anesthesiologist, the block was considered safe and effective. Additionally, this study was a single-center study, and thoracic surgeries were performed by different groups of operators. The varying levels of operator expertise may have led to biased results.

In conclusion, preoperative thoracic paravertebral nerve block with a combination of compound betamethasone and ropivacaine can reduce acute postoperative pain and decrease the incidence of chronic postoperative pain in patients undergoing thoracic surgery. It also reduces the incidence of postoperative adverse reactions, making it a superior adjuvant for thoracic paravertebral nerve block with LAs, worthy of clinical promotion and application.

## Acknowledgments

This research was funded by National Natural Science Foundation of China (No. 81701106) and the Subsidized Projects of Nantong Science and Technology Bureau (MSZ2024092).

## Author contributions

**Conceptualization:** Yibin Qin.

**Data curation:** Mengru Cui.

**Formal analysis:** Dingying Ge.

**Investigation:** Mengru Cui, Qing She.

**Methodology:** Mengru Cui, Qing She.

**Project administration:** Mengru Cui, Qing She.

**Software:** Dingying Ge.

**Supervision:** Yibin Qin.

**Validation:** Yibin Qin.

**Visualization:** Dingying Ge.

**Writing – original draft:** Cui’e Lu.

**Writing – review & editing:** Cui’e Lu.
